# Study protocol: the OptiScreen-Study: optimized psycho-oncological care—from screening to intervention

**DOI:** 10.1007/s00432-022-04368-5

**Published:** 2022-09-27

**Authors:** Tanja Zimmermann, Lara Dreismann, Viktoria Ginger, Marit Wenzel, Beate Hornemann, Franziska Dietzsch, Charis Sura, Martin Bornhäuser, Anja Mehnert-Theuerkauf, Svenja Heyne, Ines Gockel, Florian Lordick, Anke Franzke, Jürgen Weitz, Arndt Vogel

**Affiliations:** 1 Department of Psychosomatics and Psychotherapy, Hannover Medical School, Hannover, Germany; 2 National Center for Tumor Diseases (NCT/UCC), University Hospital Carl Gustav Carus, TU Dresden, Dresden, Germany; 3 Department of Medical Psychology and Medical Sociology, University Medical Center Leipzig, Leipzig, Germany; 4 Department of Visceral, Thoracic and Vascular Surgery, University Medical Center Leipzig, TransplantLeipzig, Germany; 5 Department of Medicine (Oncology, Gastroenterology, Hepatology, Pulmonology, and Infectious Diseases), University Cancer Center Leipzig (UCCL), University of Leipzig Medical Center, Leipzig, Germany; 6 Comprehensive Cancer Center, Hannover Medical School, Hannover, Germany; 7 Department of Visceral, Thoracic and Vascular Surgery, University Hospital Carl Gustav Carus, Technische Universität Dresden, Dresden, Germany; 8 Department of Gastroenterology, Hepatology and Endocrinology, Hannover Medical School, Hannover, Germany

**Keywords:** Psychooncology, Screening, Psychological distress, Training for nurses, Psychooncology care

## Abstract

**Purpose:**

Adequate, needs-oriented psycho-oncological care contributes to reducing psychological distress in cancer patients and their relatives and improving quality of life. Regarding the precise determination of objective and subjective needs, there are often discrepancies in practice between the screening instrument completed by patients, the clinical impression of the treatment team, and the judgment of the psycho-oncology team.

**Methods:**

The present multicenter study “OptiScreen”, involving three German Comprehensive Cancer Centers (Hannover, Leipzig, Dresden), aims to professionalize psychosocial screening to enable targeted and needs-based allocation to psycho-oncological support. Optimization and professionalization of the screening process will be achieved by training of oncological nursing staff to improve the targeted identification of distressed patients and provide them with needs-based psycho-oncological care. The non-randomized pre-post study will include inpatients with gastrointestinal cancers from the visceral oncology centers at the three sites. First, the comparison group (CG) will be assessed of *N* = 300. After completion of nursing training, the intervention group (IG) with *N* = 600 patients will be evaluated by validated questionnaires.

**Results:**

The aim is to reduce barriers on both the patient and treatment side by promoting interdisciplinary dialogue and linking the screening with a personal consultation offer provided by the nurses, which should help to increase utilization and reduce patients' fears, shame and information deficits.

**Conclusion:**

It is not sufficient to establish a well-validated screening procedure, it also has to be feasible and useful in clinical practice. “OptiScreen” aims to improve the psycho-oncological care situation. In parallel, the study enables the professionalization of psycho-oncological care with the involvement of important professional groups (e.g. nursing) and thus aims to develop a “best practice model”.

**Supplementary Information:**

The online version contains supplementary material available at 10.1007/s00432-022-04368-5.

## Introduction

An adequate, needs-based psycho-oncological care contributes to reduce the burden of cancer patients and their relatives and to increase the quality of life (Faller et al. 2013). About half to two-thirds of outpatients and/or inpatients in Germany with various tumor entities show significant psychological distress (Mehnert et al. 2018; Peters et al. 2020), and approximately one-third of patients from in- and outpatient care facilities develop mental disorders (Mehnert et al. 2014). Previous research elicits cancer patients’ need for psycho-oncological care (Brix et al. 2008; Faller et al. 2016; Merckaert et al. 2010) and their beneficial effects on distress and quality of life (Faller et al. 2013; Kalter et al. 2018; Linden and Girgis 2012; Osborn et al. 2006; Rehse and Pukrop 2003). An implementation of psycho-oncological care for addressing psychosocial concerns of cancer patients is anchored in the S3 guideline "Psycho-oncological care of cancer patients" for Germany (Leitlinienprogramm Onkologie 2014) and in the National Cancer Plan (Bundesgesundheitsministerium 2012). As consented there, cancer patients can expect psycho-oncological support according to their needs (Herschbach and Mandel 2011). However, despite improvements in psycho-oncological care in Germany, defining the term “needs-based” remains a problem (Schalhorn and Herschbach 2016). The targeted identification of psychological distress in all cancer patients is still very incomplete and heterogeneous in clinical practice (Koehler et al. 2017). Clarification regarding the following questions is still required: Who is in need of psycho-oncological care and, above all, who determines this need and how?

It seems that neither the patient's self-assessment nor the medical team's assessment can satisfactorily explain these issues (Carlson et al. 2010). Thus, cancer patients in need of treatment and support may be overlooked. One consequence of this resulting psycho-oncological underuse is the recommendation to use screening questionnaires and thus to optimize the allocation to psychosocial measures (Schalhorn and Herschbach 2016; Stengel et al. 2021). According to a current best practice recommendation, for a successful establishment of a distress screening under routine clinical conditions, the following factors are necessary: “sufficient, trained and competent persons who actively take responsibility for screening are needed, as are detailed, standardized, and optimized procedures for implementation, evaluation, documentation, and referral” (p. 4, Stengel et al. 2021). Particularly in patients with surgical procedures, such as visceral oncology wards, there is often a lack of implementation of psycho-oncological care due to the focus on surgical procedures and somatic treatments (Burton and Parker 1997).

## Identification of psychosocial distress

Brief distress screening questionnaires should thus increase referral to psychosocial interventions (Andersen et al. 2014; Fitch et al. 2018). In accordance with the S3 guideline Psycho-oncology (Weis et al. 2022; Leitlinienprogramm Onkologie 2014), the following screening methods are recommended: Distress Thermometer (DT) (Mehnert et al. 2006a, b), Cancer Patient Stress Questionnaire (Book et al. 2011), Hospital Anxiety and Depression Scale (Herrmann-Lingen et al. 2011), and Patient Health Questionnaire (Kroenke et al. 2001) as well as Generalized Anxiety Disorder Scale-7 (Spitzer et al. 2006). However, the use of a screening procedure does not ensure that all persons screened as burdened are identified and also referred to psycho-oncological care. Often, patients are referred to psycho-oncology through their own initiative or through the initiative of a member of the medical team. This can lead to a not inconsiderable number of patients who need support not receiving it, because they may be too burdened to request it or, without a standardized approach, have difficulty finding appropriate help (Carlson et al. 2010). Furthermore, the use of screening does not seem to automatically lead to distressed patients also seeking psycho-oncological support (Funk et al. 2016). Only about 20–40% of highly distressed cancer patients make use of psychosocial services (Dilworth et al. 2014). In certified oncology centers, the utilization rate was 11% (Singer et al. 2013). Reasons for this are that patients often deny psychosocial problems and are afraid of stigmatization, "want to make it on their own" or "have to be strong", "do not want to be a burden to others—including the treatment team" or possibly do not understand the term "psycho-oncological support". In addition, there is a lack of information about the available services and unclear ideas about their benefits (Weis and Giesler 2016). In a multicenter, cross-sectional study in Germany by Faller et al. (2016) with 4020 cancer patients with different tumor entities only 38% of patients reported feeling sufficiently informed about psycho-oncological care. Furthermore, 36% requested more information about psycho-oncological support services. Moreover, insufficient communication between patients, physicians and nurses can be the cause. Thus, the implementation of a screening often has no influence on the use of psycho-oncology support (Braeken et al. 2013). Challenges of screening include overestimation or underestimation of mental distress as well as acceptance of the screening instrument. Furthermore, the mere use of a screening instrument cannot reliably measure emotional distress (Mitchell et al. 2011). However, if the screening (DT) is combined with a personal conversation and referral offers, the use of psycho-oncology support also increases (Carlson et al. 2010). In addition, the confidence as well as the skills of the clinician also have a positive impact on successful screening (Mitchell et al. 2011).

Moreover, there is a significant number of patients who seek support in the absence of mental comorbidity (Dilworth et al. 2014). The problem in determining need between subjective and objective need can lead to a patient not receiving support despite subjective suffering according to objective criteria (screening), whereas a person needs support according to objective criteria but does not express a subjective need for support. Standardized screening instruments usually cannot solve this problem.

Thus, despite the recommendation to provide needs-based psycho-oncological care as an integral part of comprehensive cancer treatment (Herschbach and Mandel 2011), which is obligatory for oncological centers and organ cancer centers after certification by the German Cancer Society, the on-site reality of psycho-oncological care structures is very heterogeneous and incomplete (Weis and Giesler 2016). Moreover, it should be taken into account that a considerable number of patients do not complete the screening at all. Nevertheless, there seems to be a need for optimization when it comes to implementation in practice. The instrument alone does not seem to be sufficient; it also requires "manpower" (Jacobsen and Jim 2008; Stengel et al. 2021). Barriers on the part of the treatment team might contribute to this problem, due to a lack of knowledge and skills regarding the use of a screening and lack of confidence in communication during the screening process (Mitchell et al. 2008).

The degree of implementation of screening procedures worldwide and also in Germany is very heterogeneous and often associated with a considerable organizational recording and evaluation effort. Screening should be quick, feasible, and easy to use in everyday routine. However, it should lead to rapid identification of patients in need of care and thus allow for high accuracy and simple evaluation, ensuring timely referral for psycho-oncology care and further clarification of indications. Improvements in care can only be achieved if additional staffing is provided. However, there are not enough resources available, especially in terms of highly qualified psycho-oncologists, to perform screenings. According to Mitchell (2013), screening reduces distress and improves quality of life only if barriers are removed. Significant barriers were identified as: lack of qualification and support of the treatment team, low acceptance by treatment providers, and lack of linkage between screening results and treatment.

The question arises whether other professional groups can also be trained in the implementation of a psychosocial screening and thus contribute to patients moving from screening to intervention. The S3 guideline on psycho-oncology also emphasizes the multiprofessional cooperation of different occupational groups (Stengel et al. 2021). However, nurses are less perceived in psycho-oncological care than other professional groups (Dautel 2015). There is close and frequent contact between patient and nurse, during which psychosocial stress is also discussed. However, nurses often avoid these discussions for fear of not behaving appropriately or not considering these discussions as a task area (Dautel 2015). This is not due to a lack of willingness on the part of the nurses to engage in such discussions, but rather to workload compression, lack of time and also a lack of competence, particularly in communication (Dautel 2015).

## The present study

Based on these preliminary findings, the primary objective of the study was derived: to optimize and professionalize the psychosocial screening process by training oncology nurses and developing an interdisciplinary care algorithm, thereby improving the targeted identification of mental distressed patients and the referral to psycho-oncology care as needed.

**Fig. 1:**
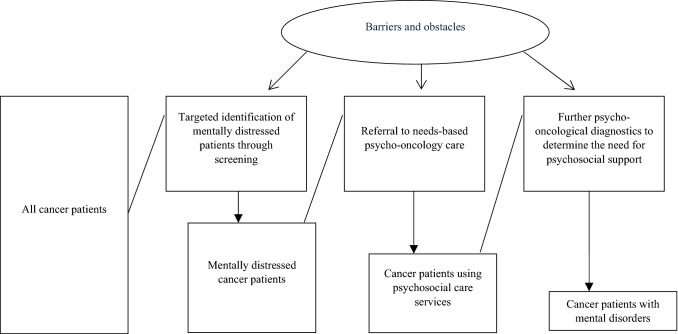
Model for targeted identification of mentally distressed patients and referral to needs-based psycho-oncology care

The postulated model of this study (Fig. 1) for the precise identification of distressed patients and for the provision of needs-oriented psycho-oncological care takes various processes into account. From all cancer patients, the mentally distressed patients have to be identified with the help of a screening. In the next step, these mentally distressed patients must also be provided with psycho-oncological care in line with their needs. The last step is to check whether the patients identified by the screening need psychosocial support through further psycho-oncological diagnostics—also for the diagnosis of mental disorders. In addition, barriers and obstacles need to be identified.

In this context, one question is whether current screening is too unspecific in terms of identifying mentally distressed patients. On the other hand, there is also the question of what a "screener"—i.e., the person who carries out the screening—must be able to do. Important questions in this context are: How is the screening introduced? How is the screening evaluated? How are the results to be interpreted? What support does a patient need? When does the patient need to be consulted? "OptiScreen" aims to address precisely these issues and thus develop a "best practice model" based on the needs of cancer patients and the available resources. "OptiScreen" aims to train oncology nurses as "screeners" and, therefore, reduce personal and structural barriers in the screening process.

## Study objectives

The following questions and objectives are to be examined:

1. Acceptance of psycho-oncological screening: does the implementation of existing screening by trained oncological nurses lead to a higher acceptance of psychosocial screening among patients and the medical treatment team?

2. Targeted, needs-based referral to psycho-oncology: Does existing screening by trained oncology nurses lead to more targeted psychosocially distressed patients (identified by the screening cut-off value) to a psycho-oncological consultation? Are these patients mentally distressed (further psycho-oncological diagnostics)? Does the psycho-oncology team also report more targeted referrals of distressed patients to psycho-oncology?

3. Patient competence and satisfaction: Does the implementation of the screening by trained oncological nurses increase the knowledge and competence of the patients about psycho-oncological services, their acceptance and satisfaction?

4. Competence and satisfaction of the nursing staff: Does the implementation of the screening by trained oncological nursing staff increase the competence of the nursing staff, reduce possible fears or insecurities in contact with cancer patients and increase satisfaction?

5. Acceptance of psycho-oncological offers: does interdisciplinary collaboration lead to greater acceptance of psycho-oncological services and a reduction in obstacles and barriers?

6. Development of a tested training concept for oncological nurses to professionalize psychosocial screening.

7. Acceptance, satisfaction, and feasibility of training from the nursing perspective.

The OptiScreen-Training aims to improve (a) the identification of distressed patients and their referral to psycho-oncological support, (b) the patients’ satisfaction with the screening process and information regarding support options, (c) nurses’ acceptance of performing the screening, (d) nurses’ confidence and satisfaction regarding communication during the screening process, and (e) interdisciplinary communication between nurses and the psycho-oncological team.

The aim is to reduce barriers in psycho-oncological care for patients, and practitioners by developing a “best practice-model” for the screening process through training.

## Materials and methods

### Participants

The study population includes inpatients of the visceral oncology centers at the three sites. The following inclusion criteria are defined: (a) diagnosis of a malignant solid tumor in the context of a visceral oncological disease (according to the survey form of Onkozert (2018): intestine, pancreas, stomach, liver, esophagus); (b) a minimum age of 18 years; (c) cognitive ability to consent to study participation. The following exclusion criteria are established: severe physical, cognitive, and/or language limitations (not be able to fill in questionnaires in German language).

All nurses at the visceral oncology centers at the three sites are expected to participate in the training during their working hours. Nurses were offered the opportunity to complete an evaluation before and after the training.

In addition, psycho-oncologists will also be asked about their experience with the overall screening process before and after the training, as well as patient distress.

### Design

**Fig. 2:**
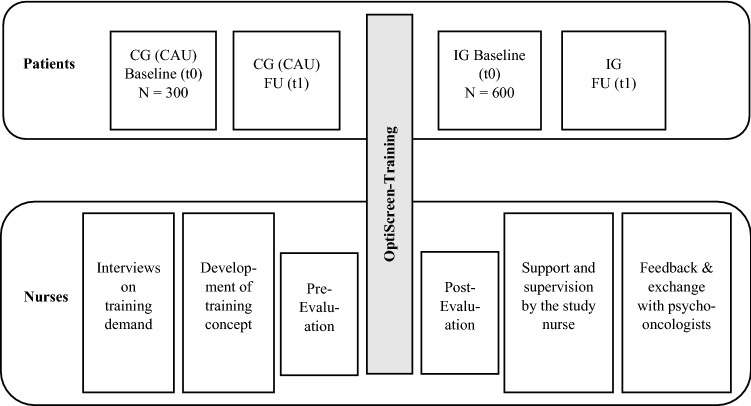
Study design. CG = Comparison group; CAU = Care as usual; IG = Intervention group, t1 = 3 months follow-up (FU)

The study design is uncontrolled before and after pilot study. Measurement time points are a baseline measurement during the inpatient stay (t0) and a 3-month follow-up (t1 postal or online). As a comparison sample (CG, *N* = 300), screening data are collected 6 months prior to the introduction of the "OptiScreen-Training" on the same wards as after the introduction of the optimized screening, which thus corresponds to a care-as-usual (CAU) condition (Fig. 2). Care-as-usual refers to the identification of mentally distressed patients in the same way as is customary in their respective wards. Advantages of this approach are cost efficiency and practicality. After completion of the OptiScreen-Training, an additional *N* = 600 patients (intervention group, IG) will be surveyed at t0 and t1 (Fig. 2).

Positive ethics votes have been received from Hannover Medical School (8478_BO_K_2019), University Medical Center Leipzig (274/19-lk) and University Medical Center Dresden (EK 459,102,019).

### Sample size and power calculation

Based on the primary cases in the centers, *n* = 200 patients per center will be included in the IG over a recruitment period of 24 months. For the CG, *n* = 100 patients per center will be recruited. Thus, a study population of *N* = 300 patients in the CG and *N* = 600 patients in the IG can be assumed.

For the primary analysis of the main outcome variable (mental distress of patients), small to medium effects of the IG compared to the CG are assumed. Assuming an effect size of 0.3, a sample size of *n* = 190 evaluable persons each in CG and IG are required for the primary analysis to be able to prove the assumed effect with a power of 80% (G*Power). With a dropout of 50%, a total of *n* = 270 patients per group (CG and IG) resulting in a total of *N* = 540 must be recruited at t0.

For the study, the case numbers of the participating oncology centers result in *N* = 1,533 cases per year. For the recruitment period of 24 months, a total pool of *N* = 3,066 can thus be expected. Thus, after subtracting the expected dropout rate of 50%, *N* = 1,533 patients can potentially be recruited for the study. Thus, the target sample size of *N* = 600 (IG) can be considered feasible. Since the CG will be recruited before the IG, the targeted sample size of* N* = 300 can also be considered feasible.

### Procedure and recruitment

#### Patients

Cancer patients will be recruited via the respective visceral oncology centers of the three study sites. The eligibility criteria will be assessed by the study coordinators. If patients decline to participate in the study, a 3–5 min interview will be conducted to capture reasons for refusal, previous screening and psycho-oncology experiences, and information about the current disease. The participants receive the same questions in the questionnaire. If the inclusion criteria are fulfilled and the patient is interested in participating in the study, a detailed explanation is given in a personal interview (informed consent) after the initial contact with the patient. If the person declares their willingness to participate, a signed consent form is obtained.

In addition to the screening, the patients receive a questionnaire package (approximately 30–40 min) at t0 and t1 (Table 1). Furthermore, demographic, medical and treatment-related data are collected. The patients receive the questionnaires in person (at t0 during the inpatient stay) and by mail or e-mail as an online survey via link (at t1). The online questionnaire will be provided via a secure online survey platform (UniPark). Completed questionnaires will be collected by the study nurse and the study coordinators. If questionnaires are not received at t1, a reminder will be sent (by mail or e-mail) after 2 weeks.

**Table 1:** Measurement instruments

Questionnaire	CG t0	CG t1	IG t0	IG t1
*Basic data*				
Demographic, medical and treatment-related data	x	x	x	x
*Screening*				
Distress (NCCN-DT)	x	x	x	x
Corona-DT	x	x	x	x
Knowledge about psycho-oncology (OptiScreen Questionnaire)	x	x	x	x
*Psychological functioning*				
Depression (PHQ-9)	x	x	x	x
Anxiety (GAD-7)	x	x	x	x
Quality of life (SF-8)	x	x	x	x
Fear of cancer recurrence (PA-F-KF)	x	x	x	x
Health literacy (HLS-EU-Q16)	x	x	x	x
Support needs (SCNS-SF34-G)	x	x	x	x
Social support (ISSS)/SSUK-8)	x	x	x	x
Body image (BIS)	x	x	x	x
Patient activation (PAM-13D)	x	x	x	x
Satisfaction with care (REPERES-33-G)	x	x	x	x
Relationship satisfaction (OMI-D)^a^	x	x	x	x
Sexuality (SBQ-G)	-	-	x	x

*Notes* CG = comparison group; IG = intervention group; NCCN-DT = National Comprehensive Cancer Network Distress Thermometer (Mehnert et al. 2006a, b); Corona-DT = Adapted version of the DT to measure psychological distress from the Covid-19 pandemic, PHQ-9 = Depression module of the Patient Health Questionnaire (Kroenke et al. 2001); GAD-7 = Generalized Anxiety Disorder Questionnaire (Spitzer et al. 2006); SF-8 = Short Form-8 Health Survey (Ware et al. 2001); PA-F-KF = Fear of progression Questionnaire, short version (Mehnert et al. 2006a, b); HLS-EU-Q16 = European Health Literacy Questionnaire (Sorensen et al. 2013); SCNS-SF34-G = Supportive Care Needs Survey, short form (Sklenarova et al. 2015); ISSS/SSUK-8 = The Illness-specific Social Support Scale (Ullrich and Mehnert 2010); BIS = Body Image Scale (Hopwood et al. 2001); PAM-13D = Patient Activation Measure (Brenk-Franz et al. 2013); REPERES-33-G = Recherche Evaluative sur la Performance des Réseaux de Santé, German short version (Defossez et al. 2007); QMI-D = Quality of Marriage Index (Zimmermann et al. 2015); SBQ-G = Sexual Behavior Questionnaire (Müller and Gensch 2003)

^a^only for patients in relationships

All patients are given a code to ensure anonymity. All study documents will be stored in locked cabinets, and data will be stored in password-protected files accessible only to the study team.

The comparison sample (CG) is collected at baseline as a care-as-usual condition. The survey will take place before the OptiScreen-Training is conducted.

To survey the intervention group (IG), after conducting the oncology nursing training, the trained nurses are assigned per site to conduct the screening. Their task is to screen all inpatients for psychosocial distress using the screening instrument, e.g., the Distress Thermometer (Mehnert et al. 2006a, b). The nurses evaluate the screening and also consider the clinical impression of the treatment team. When performing the screening, the nurses take into account the aspects taught in the training regarding framework conditions and obstacles. In case of elevated distress, further psycho-oncological diagnosis and care is provided by the psycho-oncology team, which in turn provides feedback on the patient's psychological distress to the medical treatment team in a feedback loop. If there is no to low stress, a low-threshold information offer (e.g., flyer) is made.

#### Psycho-oncology team

To obtain a validated external assessment of patients' psychological distress and support needs, information will be collected by the psycho-oncology team after the initial consultation with patients (assessment sheet psycho-oncological consultation). In addition, the psycho-oncology team will be surveyed after the OptiScreen-Training has been conducted to assess acceptance, satisfaction, and changes in nurses' attitudes and behaviors regarding the psycho-oncology screening process.

#### Nurses

Nursing staff will also be surveyed on satisfaction, feasibility and acceptance of the OptiScreen-Training via questionnaire (paper–pencil). The pre-questionnaire for the nurses is completed two weeks prior to the training, the post-questionnaire immediately after the OptiScreen-Training. Nurses choose a personal code for anonymity to compare the pre- and post-questionnaires.

#### The “OptiScreen-Training”

**Fig. 3:**
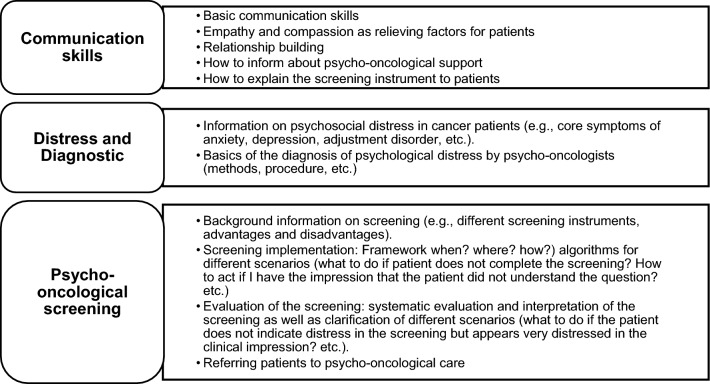
Modules of the OptiScreen-Training

The concept of the OptiScreen-Training is based on qualitative analyses of nurses training needs (Dreismann et al. 2022) as well as literature, evidence-based examples, and a workshop with experienced psycho-oncologists. To achieve a good fit of the training and the needs of nurses, we conducted interviews with 15 experts on nursing from all three study locations to determine barriers, requirements and major questions (Dreismann et al. 2021, 2022). The OptiScreen-Training consists of three main thematic modules (Fig. 3) that can be taught in one day or on three different dates, each lasting about 2 h as face-to-face sessions. Module 1 focuses on psycho-oncological care and possible mental disorders in cancer patients. Module 2 addresses psychological distress and the screening process. Module 3 aims to train communication during the screening process and teaches basic approaches to self-care. All modules include theoretical knowledge transfer as well as practical exercises such as role plays, group-work, case discussions and take place in groups of 4–15 nurses. All trainings are conducted by trained psychologists with expertise in psycho-oncology and are recorded to ensure comparability. The trainings are conducted on-site at the three study sites. To maintain the learning effect of the training, concepts such as booster methods (e.g., booster session 1 year after the initial training) and refresher techniques (e.g., postcards) are developed to remind nurses to integrate the screening into their daily work.

For the successful implementation of the screening process after training, a local study nurse supports the nursing staff at all three sites in the implementation and evaluation of the screening as well as the referral to psycho-oncology. With the help of the study nurse, the nurses try to overcome barriers and obstacles of the patients regarding the screening through advisory exchange with each other. The study nurse is in direct exchange with the psycho-oncology team. She is also a multiplier for the optimized screening in the medical team (e.g. nurses, casemanagement, physicians) and answers questions regarding the process or specific cases. The study nurse tries to reduce barriers and obstacles in communication and utilization of the screening and to optimize the implementation in the daily clinical routine.

#### Measurement

To measure the study objectives the following outcomes should be captured: (a) psychological distress of patients, (b) utilization of psycho-oncological support, (c) information about psycho-oncological services, (d) treatment satisfaction, (e) acceptance and practicability of OptiScreen-Training by caregivers, (f) assessment of access to and utilization of support services, barriers and facilitating factors for utilization of psycho-oncological services. A list of measurement instruments is presented in Table 1.

#### Primary outcomes

Mental distress of patients (identified as mentally distressed by optimized screening, receive a psycho-oncology consultation and further diagnosis by psycho-oncology verifies mental distress) is the primary outcome. To assess distress, the German version of the National Comprehensive Cancer Network Distress Thermometer will be used (Mehnert et al. 2006a, b). General distress is measured on a visual analogue scale ranging from 0 = no distress to 10 = extreme distress. A score of 5 is internationally recommended as an indicator that a patient is distressed and needs support (National Comprehensive Cancer Network 2020). Further 36 potential causes of distress that are grouped in five subscales, i.e., (1) practical problems, (2) family problems, (3) emotional problems, (4) spiritual/religious concerns, (5) physical problems and an open answer option for possible other problems are assessed using the problem checklist (yes/no).

#### Secondary outcomes

Information about psycho-oncology services, treatment satisfaction, patient competence and quality of life are secondary outcomes as well as acceptance and practicability of the OptiScreen-Training, assessment of access to and utilization of support services, barriers and facilitating factors for the utilization of psycho-oncological services.

#### OptiScreen questionnaire

An individual questionnaire (OptiScreen questionnaire) was developed to assess patients' experiences with screening and psycho-oncology. Questions address psychological support prior to disease, as well as experiences with psycho-oncologists during current hospitalization or need for further psychological support after discharge (use of support and need). Attitudes toward psycho-oncology, barriers, and satisfaction with support services were also recorded (satisfaction and barriers). In addition, experiences with caregivers regarding information about psycho-oncology, brochures received, and the screening questionnaire are asked (information and screening).

#### Assessment sheet psycho-oncological consultation

For psycho-oncologists, a questionnaire and documentation sheet ("assessment sheet psycho-oncological consultation") is prepared, which is to be completed by the psycho-oncologist after the psycho-oncological consultation in the hospital. The assessment sheet contains information about the patient and duration of the psycho-oncological consultation, information about the cancer diagnosis and treatment, level of information of the patient about psycho-oncology (information provided by the medical treatment team, receipt of a flyer about psycho-oncology, knowledge about the upcoming psycho-oncological consultation), patient's need for psycho-oncological care, patient's competence to recognize own need for psycho-oncological support, patient's openness to psycho-oncological support, patient's psychological distress/illness as well as previous psychological illnesses, type, extent and quality of psycho-oncological exchange with current oncological treatment team, environmental factors (organization, materials, facilities, patient's accessibility, documentation, etc.) to ensure psycho-oncological support, degree of burden of the psycho-oncologist by the psycho-oncological consult. In addition, the patients' symptom and stress areas will be evaluated as a third-party assessment in comparison to the patient survey. Synchronous to the patient survey, short versions or adapted parts of the questionnaires will be used as external evaluation (Table 1).

#### Training evaluation questionnaire for nurses

The training evaluation questionnaire includes sociodemographic data, questions about current knowledge and experience with psycho-oncology and the screening measures. Pre-training expectations and attitudes toward screening are asked. Questions are answered on a five-point Likert scale (1 = not at all to 5 = very much).

Additionally the post-evaluation contained the German Training Evaluation Inventary (Ritzmann et al. 2014) which shows good internal consistency (Cronbachs *α* = 0.73). It includes five scales on training outcomes dimension (perceived fun, perceived difficulty, perceived usefulness, knowledge acquisition, attitude) and five scales on training design dimension (problem-based learning, activation of prior knowledge, demonstration, application, integration). The respective items can be answered on a five-point Likert scale (0 = I disagree to 4 = I strongly agree). High sum scores represent a higher rating of the respective underlying dimension.

#### Statistical measures

First, the primary and secondary outcomes as well as all patient characteristics are analyzed descriptively together and separately for the two groups (means, standard deviations, frequencies). The primary outcome, psychological distress, is analyzed by ANCOVA (analysis of covariance) including variables such as tumor stage, age, and gender as covariates. This usually even increases the power of an ordinary *t* test. Missing values are replaced using multiple imputation. In sensitivity analyses, the results of the primary analysis are checked for consistency. Differences in secondary outcomes are evaluated between intervention and comparison groups or between appropriate subgroups analogous to the primary analysis using univariate or multivariate procedures or appropriate regression models. Depending on the distribution of the target parameter, binary or ordinal logistic mixed model regressions and linear mixed model regressions will be used. Before starting the analysis, the exact procedures are defined in a statistical analysis plan. Moderator as well as mediator analyses are used to identify barriers to take-up, as they can test complex relationships between several variables in the form of a priori models. The evaluation of the training will be analyzed using *t* tests (pre and post).

To control for possible selection effects that could lead to sampling bias, reasons for non-participation and sociodemographic and medical data of the non-participants will be analyzed. In this way, possible selection effects can be identified and the interpretation of results may be adjusted as necessary. Possible confounding variables are analyzed in inferential statistical analyses (e.g., regressions) using important covariates (age, gender, tumor entity, stage) as well as other theory-based influencing factors, e.g., physical comorbidities controlled.

Missing values due to drop-outs are replaced and can be predicted by means of multiple imputation, in that for the missing values, estimated values are used, which are calculated by distributing different predictors. This is done by taking all available relevant information of the data set into account and including random errors. The advantage is a low loss of information, since all variables remain included in the model. In addition, standard errors, which are moreover caused by the multiple repetition of the estimation process are randomly and realistically calculated and included.

Qualitative methods such as content analysis according to Mayring and Fenzl (2019) are used for the evaluations of the free responses.

## Discussion

Approximately half to two-thirds of cancer patients report significant distress (Mehnert et al. 2018; Peters et al. 2020), while research suggests that 35–40% of patients would already benefit from basic psychosocial interventions, such as provision of information (Chiles et al. 1999). Adequate, needs-based psycho-oncological care contributes to reducing the distress of cancer patients and their relatives and to improving the quality of life (Faller et al. 2013). To ensure patient-oriented psycho-oncological care, patients who are mentally distressed and in need of care must be referred to psycho-oncology in a targeted manner (Gotz et al. 2019). In most cases, referral to psycho-oncology is made through a consultation of the medical treatment team and thus presupposes an approximately reliable assessment of the need for care by the medical treatment team. In clinical practice, however, there are often different assessments between the medical, nursing and psycho-oncological teams with regard to the psychological burden and the psycho-oncological care needs of the patients.

Within the framework of this multicenter study, a newly designed training of nursing staff ("OptiScreen-Training") on psychological stress and psycho-oncological care is intended to increase the targeted identification of psychologically stressed patients in need of treatment, to increase the referral of these patients to psycho-oncology and to improve interdisciplinary cooperation. Furthermore, the training should lead to a better acceptance of psycho-oncological screening among medical staff and patients. The training should help to reduce fears or uncertainties in contact with psychologically stressed cancer patients and to increase the competence and satisfaction of the nursing staff in dealing with the psychological stress of oncological patients. Moreover, barriers on the part of the patients as well as on the part of medical treatment are to be reduced and a productive interdisciplinary exchange is to be promoted. Further goals are the reduction of unpleasant emotions among patients, such as shame and fear, as well as the reduction of information deficits about the psychological burden of cancer. In addition, the information content about psycho-oncological services of those affected is to be increased. Furthermore, the use of patient-oriented psycho-oncological treatments is to be increased and an increase in the treatment satisfaction and the quality of life of patients is to be achieved. To our knowledge, this is the first study to examine systematic training of nurses on psycho-oncology screening and to use a study nurse to implement the training content into daily routine.

The aim of the study is to achieve a professionalization of the clinical impression of the treatment team regarding the mental distress of cancer patients as well as to increase the acceptance of the screening and to provide psychologically highly stressed patients with support according to their needs. In summary, the findings of this multicenter study should contribute to the improvement of interdisciplinary cooperation, improve the need- and target-oriented allocation of psychologically stressed patients to psycho-oncological care and thus develop a "best practice model" of an interdisciplinary care algorithm.

## Supplementary Information

Below is the link to the electronic supplementary material.

Supplementary file1 (DOCX 30 kb)

## Data Availability

The datasets generated during the current study are available from the corresponding author on reasonable request.
